# Dynamic spherical harmonics approach for shape classification of migrating cells

**DOI:** 10.1038/s41598-020-62997-7

**Published:** 2020-04-08

**Authors:** Anna Medyukhina, Marco Blickensdorf, Zoltán Cseresnyés, Nora Ruef, Jens V. Stein, Marc Thilo Figge

**Affiliations:** 1 Applied Systems Biology, Leibniz Institute for Natural Product Research and Infection Biology – Hans Knöll Institute (HKI), Jena, Germany; 2 Institute of Microbiology, Faculty of Biological Sciences, Friedrich Schiller University Jena, Jena, Germany; 3 Department of Oncology, Microbiology and Immunology, University of Fribourg, Fribourg, Switzerland; 4 Center for Sepsis Control and Care (CSCC), Jena University Hospital, Jena, Germany; 5 Present Address: Center for Bioimage Informatics, St. Jude Children’s Research Hospital, Memphis, TN USA

**Keywords:** Computational biology and bioinformatics, Computational biophysics, Signal processing, Time series, Cell migration

## Abstract

Cell migration involves dynamic changes in cell shape. Intricate patterns of cell shape can be analyzed and classified using advanced shape descriptors, including spherical harmonics (SPHARM). Though SPHARM have been used to analyze and classify migrating cells, such classification did not exploit SPHARM spectra in their dynamics. Here, we examine whether additional information from dynamic SPHARM improves classification of cell migration patterns. We combine the static and dynamic SPHARM approach with a support-vector-machine classifier and compare their classification accuracies. We demonstrate that the dynamic SPHARM analysis classifies cell migration patterns more accurately than the static one for both synthetic and experimental data. Furthermore, by comparing the computed accuracies with that of a naive classifier, we can identify the experimental conditions and model parameters that significantly affect cell shape. This capability should – in the future – help to pinpoint factors that play an essential role in cell migration.

## Introduction

**Figure 1:**
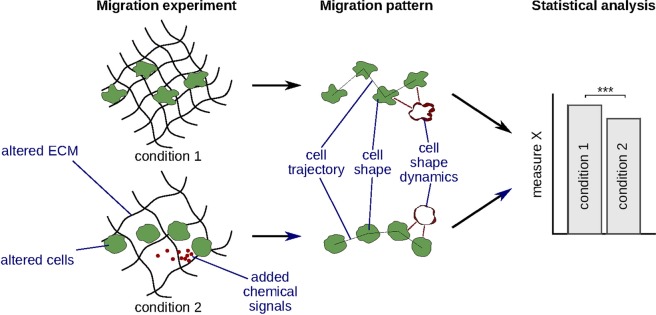
Analysis and quantification of migration patterns can help to study cell migration mechanisms. First, a migration experiment is performed, which involves altering the migrating cells (genetic or chemical modification, choosing cells of a different type), altering the extracellular matrix (ECM), or adding chemical signal to the extracellular environment. Next, cell migration patterns are analyzed and quantified. Finally, one or more quantitative measures derived from these patterns are statistically compared between conditions to detect significant differences.

A cell’s migration behavior depends on the state of the cell, extracellular environment, and signals from other cells^1^. We can study the mechanisms of cell migration by, e.g., knocking out a certain gene or altering the structure of the extracellular matrix (ECM) and testing whether these changes affect cell migration patterns, such as cell trajectory, shape, or shape dynamics (Fig. 1). To compare migration patterns in an objective and statistically sound way, they have to be automatically analyzed and quantified^2^. Whereas both cell trajectories^3,4^ and cell shape^5,6^ can be quantified with a multitude of available methods, the analysis of shape dynamics – especially in 3D – received considerably less attention.

When analyzing cell shape in a static fashion, we look at just one snapshot of the cell’s migration history. Depending on how we choose this snapshot, we may either miss important differences in cell shape – e.g., if cells transiently appear similar but have different migration patterns – or detect spurious differences – e.g., if cells occur in different phases of the same migration pattern. Even averaging cell shape descriptors over time^7^ may not always be sufficient to distinguish some migration patterns, for example when all cells evolve through similar phases of cell shape but different cells do this with different frequencies (Fig. S1)^8^. To distinguish such details of migration behavior we need dynamic shape analysis that takes into account relative changes in cell shape between consecutive time points.

While such dynamic shape analysis has been done in 2D^8–11^, 3D shape descriptors have not been applied to characterize and compare the full dynamic migration patterns of cells. The ultimate goal, however, is to understand how cells migrate in living organisms^12^. Due to advances in intravital microscopy^13–15^, we have increasingly more 3D + time data of cells migrating *in vivo* and we should exploit the potential of 3D methods to analyze these data^16^.

Although there are many simple shape descriptors that can be applied in 3D (e.g., solidity, ellipticity, prolateness), only relatively complex ones can reveal the fine details of the cell shape and classify between relevant spatial patterns while ignoring random shape variations^7^. One especially popular and promising approach involves spherical harmonics (SPHARM)^17–19^. SPHARM is a 3D extension of a Fourier analysis, where an arbitrary shape function is expanded on a sphere using a set of orthogonal spherical functions as a basis. This approach was shown to be effective for characterizing the shape of proteins^20,21^, red blood cells^22,23^, brain structures^19,24,25^, as well as migrating cells^7,26–28^. In the contexts of cell migration analysis, SPHARM have been applied to identify phases of amoeboid cell motion^26–28^ and to classify shapes of migrating cells based on SPHARM spectra averaged over time^7^. Therefore, SPHARM descriptors represent an ideal first candidate to be extended for dynamic 3D shape analysis.

In this proof-of-principle study, we investigate, whether the use of dynamic shape descriptors can improve classification between migration patterns of cells. We extend the SPHARM analysis by computing dynamic SPHARM descriptors, combine both descriptors with a support-vector-machine classifier, and compare their ability to distinguish between migration patterns of cells in synthetic and experimental data.

## Materials and Methods

To study the use of dynamic SPHARM for classifying migrating cells, we analyzed two types of input data: synthetic cells generated with an in-house developed cell migration simulator (CMS), and T cells visualized with intravital microscopy. For each cell, we extracted cell surfaces at various time points and transformed them into a static or dynamic SPHARM feature vector. We then used the computed feature vectors to classify cells according to their migration behavior.

### Cell migration simulator

**Figure 2:**
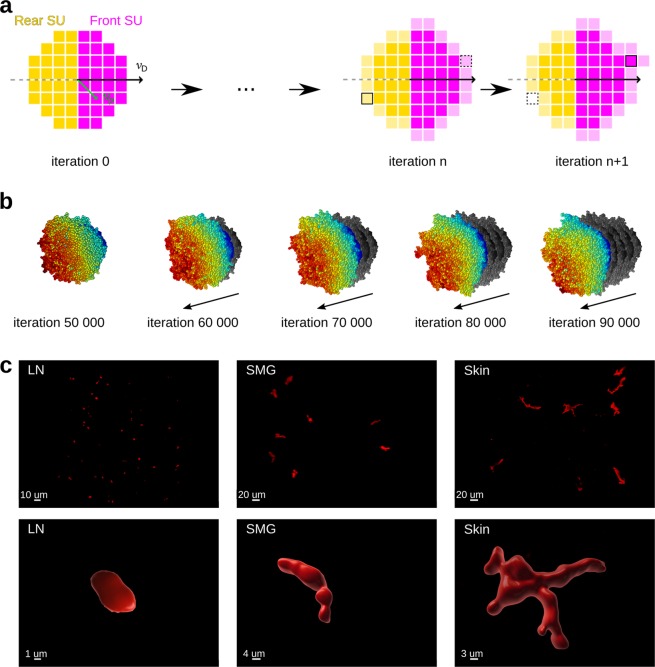
Synthetic and real migrating cells analyzed in this study. (**a**) Schematic overview of a 2D version of the cell migration simulator; the simulation starts with a spherical cell consisting of the pixel-based spatial units (SU); for each SU, we compute a position vector $\overrightarrow{{v}_{P}}$ relative to the center of mass of the cell; we randomly choose the migration direction $\overrightarrow{{v}_{D}}$ and define the cell’s front and rear perpendicularly to $\overrightarrow{{v}_{D}}$; at each iteration, we take one SU from the rear surface and move it to the front, which causes the cell to shift its center of mass; pale yellow SU indicate the candidates for removal, whereas pale purple SU indicate the free positions at the cell front. (**b**) Example of a cell generated with the 3D version of the cell migration simulator; gray shades designate the cell positions at previous time points. (**c**) Upper row: Deconvolved images of CD8^+^ T cells migrating in a lymph node (LN), submandibular salivary gland (SMG) and skin shown in Maximum Intensity Projection (MIP) mode; Lower row: individual representative cells in higher magnification zoom-in from the same groups as in the upper row in 3D Surface Rendering mode in Imaris.

To generate synthetic migrating cells, we used our previously developed cell migration simulator (CMS)^29^. In CMS, each cell consists of a set of grid-based spatial units (SU), and the cell migration in 3D is simulated by iteratively moving SU from the rear of the cell to the front (Fig. 2a).

The simulation starts with a spherical cell with the center of mass at position ${p}_{init}$ and a randomly chosen migration direction $\overrightarrow{{v}_{D}}$ (Fig. 2a). Each SU of the cell receives a position vector $\overrightarrow{\,{v}_{P}}$, which is used to compute the relative SU position *P* as the dot product between the normal vectors $\overrightarrow{{v}_{P}}$ and $\overrightarrow{\,{v}_{D}}$: $P=\overrightarrow{{v}_{D}}\cdot \overrightarrow{{v}_{P}}\in [-1,1]$. The value of *P* defines whether the corresponding SU belongs to the cell’s rear or front: for all front SU, *P* must be greater than a pre-defined front-rear threshold *FR*, whereas all SU with $\,P\le FR\,$belong to the cell’s rear. The parameter *FR* defines the fraction of the cell volume considered as the front. Thus, for $FR=0$ the front and the back have equal weights; for a negative *FR* the front is wider than the back, and for a positive *FR* the front is narrower than the back (Fig. S2a).

In addition to *FR*, the model has three further parameters: neighbor weight (NW), position weight (PW), and distance weight (DW), which define the probability of moving a specific SU from the rear to the front surface. In each iteration, an SU from the rear surface and a free position at the cell’s front are chosen randomly using Monte Carlo acceptance-rejection sampling. The acceptance probability for each SU depends on the number of neighbors *N*, position *P*, and the distance to the center of mass *D* (Fig. S2b–d) and is computed based on the rear and front scores *S*_*r*_ and *S*_*f*_: $\,{S}_{r}={|P|}^{{\rm{PW}}}\cdot {(6-N)}^{{\rm{NW}}}\cdot {D}^{{\rm{DW}}}$, ${S}_{f}={|P|}^{{\rm{PW}}}\cdot {N}^{{\rm{NW}}}$. Thus, those rear SU and free front positions have a higher probability of being selected that are closer to the cell’s migration axis (large *P*). Additionally, those SU are more likely to be removed that are further away from the cells’ center of mass (large *D*) and have fewer neighbors (small *N*), whereas the free positions at the front are preferred when they have more neighbors (large *N*).

In each iteration of the model, only one SU moves from the cell’s rear to the front. Thus, the cell shape changes only slightly even after 1000 iterations, and the cell moves a fraction of its diameter (Fig. 2b, Supplement Video S1). This means that our cell migration simulator allows us to study the changes in cell shape with high spatio-temporal resolution. Therefore, we focused on resolving the changes in cell shape rather than on following the cells for extended time intervals. We simulated each cell for 100 000 iterations and saved the coordinates of all SU every 500 iterations. We analyzed the cells starting at iteration 60 000 when the cell shape appeared sufficiently different from its initial spherical shape; this resulted in 80 time points per cell. To investigate diverse patterns of cell migration, we generated four sets of cells – one for each parameter of the model. Each set consisted of three classes corresponding to three different values of the examined parameter, and each class contained 70 cells (see section 3.1).

### Experimental data

Besides synthetic cells, we applied our analysis to intravital two-photon microscopy images of T cells migrating in the popliteal lymph node (LN), submandibular salivary gland (SMG), and skin (Fig. 2c, Supplementary Videos S2–S4). We isolated T cells from the spleen and the peripheral lymph nodes of GFP^+^ or dsRed^+^ donor mice and transferred them to recipient mice, which were subsequently infected with a virus to generate memory T cells in various organs (see Supplementary Methods for details). We visualized the T cells migrating in the LN, SMG, and skin with two-photon microscopy, and acquired six time series for each condition. All experiments were approved by the Cantonal Committee for Animal Experimentation (License 2018_22_FR) and performed in accordance with federal guidelines.

### Surface extraction

To obtain cell surfaces for SPHARM analysis, we reconstructed the surface of each cell as a triangular mesh and extracted the x, y, z coordinates of the mesh vertices. For synthetic cells, we converted the SU coordinates of each cell into a binary mask and reconstructed the cell surface using the marching cube algorithm implemented in the scikit-image library of Python^30^. To reconstruct the surfaces of T cells, we first deconvolved the microscopy images using HuygensPro 19.04 (SVI, Hilversum, Netherlands) and then segmented and tracked them using Imaris 9.3.1 (Bitplane, Zürich, Switzerland; see Supplementary Methods for details). The resulting cell shapes were saved as VRML files for further analysis. To unify the different time intervals between frames (20 seconds for LN and SMG vs. 60 seconds for the skin), we analyzed only every third time point in the tracks from LN and SMG.

### Feature extraction with spherical harmonics

**Figure 3:**
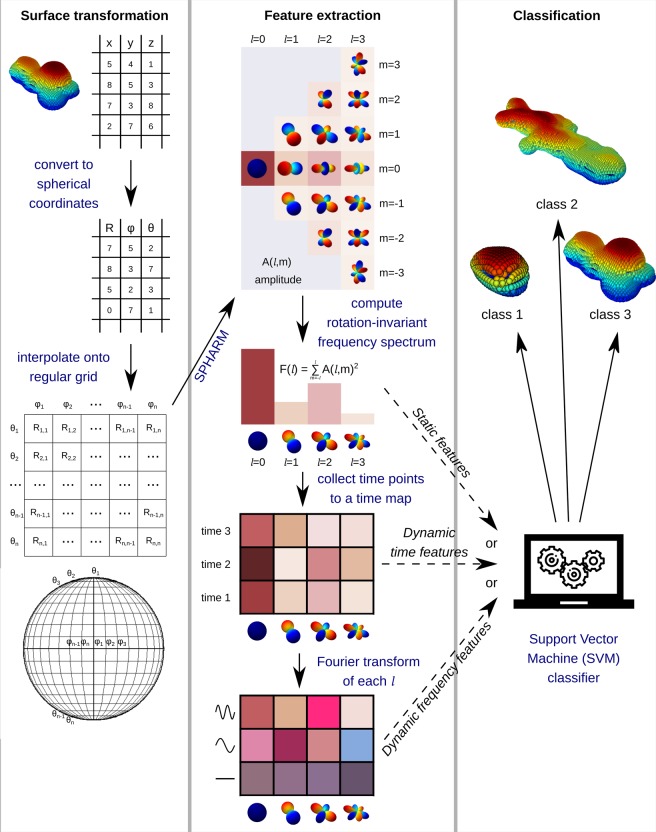
Schematic overview of the surface analysis and classification. We convert surface coordinates to a regular spherical grid, transform them with SPHARM, and compute a rotation-invariant spectrum. We use the spectrum of a single time point either as a feature vector by its own (static features) or combine it with other time points into a time map (dynamic time features). We can further convert the time map into a frequency map by Fourier transform of each degree *l* (dynamic frequency features). We then use one of the three feature vectors to classify cells according to their shape.

To analyze the cell shape and its dynamics, we decomposed the cell surface at each time point into rotation-invariant spherical harmonics. The SPHARM transform was calculated using the Python library SHTools^31^, which requires a regular N x N spherical grid as input. Such grid was obtained by converting the surface coordinates from the Cartesian to the spherical coordinate system and then interpolating them onto a 120 ×120 grid of the polar angle $\theta$ and azimuthal angle $\varphi$ (Fig. 3, left). The spherical grid was expanded into complex spherical harmonics using Driscoll and Healy’s sampling theorem^32^ implemented within SHTools. In order to obtain a rotation-invariant shape feature $F(l)$, the power $A{(l,m)}^{2}$ of each complex harmonic was computed and summed up for all orders *m* of each degree *l* (Fig. 3, middle)^33^. The first *l*_*max*_ degrees of each rotation-invariant spectrum were used to represent the shape of an individual cell at a single time point.

The rotation-invariant spectra $F(l)$ were either used to characterize the static cell shape or combined with spectra from other time points to characterize the shape dynamics (Fig. 3, middle). For both static and dynamic analysis, individual migrating cells – rather than individual time points of the same cell – served as independent observations. Thus, to obtain static shape features, we used $F(l)$ from the first time point of each cell and discarded the remaining time points. Information from different time points was combined by computing two types of dynamic features in the time and the frequency domain. To compute the features in the time domain (dynamic time features), we combined $F(l)$ from the first *T* time points of each cell into a time map $\,F(l,t)$. Being concatenated one after another, the time-ordered spectra served as an extended feature vector of size $\,{l}_{max}\cdot T$. The features in the frequency domain (dynamic frequency features) were revealed by a Fourier transform of $F(l,t)$ calculated for each *l*. This transform resulted in a frequency map $F(l,f)$ of the same size, whose values constituted another dynamic feature vector. The sizes of the three feature vectors were defined by the parameters *l*_*max*_ and *T*, which we adjusted to maximize the classification accuracy (Supplementary Methods, Fig. S3, S4).

### Shape classification

After extracting static and dynamic shape features, we classified the cells using the linear-kernel support vector machine (SVM) classifier implemented in the scikit-learn library of Python^34^ (Fig. 3, right). In order to compare the shape between different classes of cells and to identify significant shape differences, we computed the classifier accuracy between each pair of classes and compared it to the accuracy of a control classifier.

**Figure 4:**
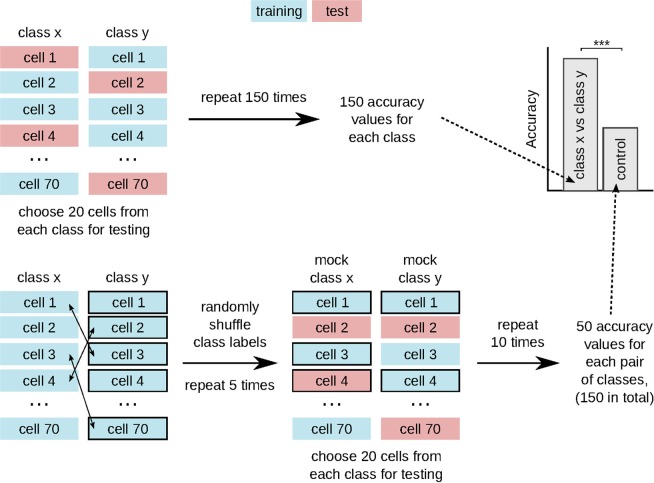
Schematic of the cross-validation scheme for synthetic data. We compute the classifier accuracy for each pair of classes using two-class classification with stratified shuffle split cross-validation. For the control group, we apply stratified shuffle split cross-validation after randomly shuffling class labels.

**Figure 5:**
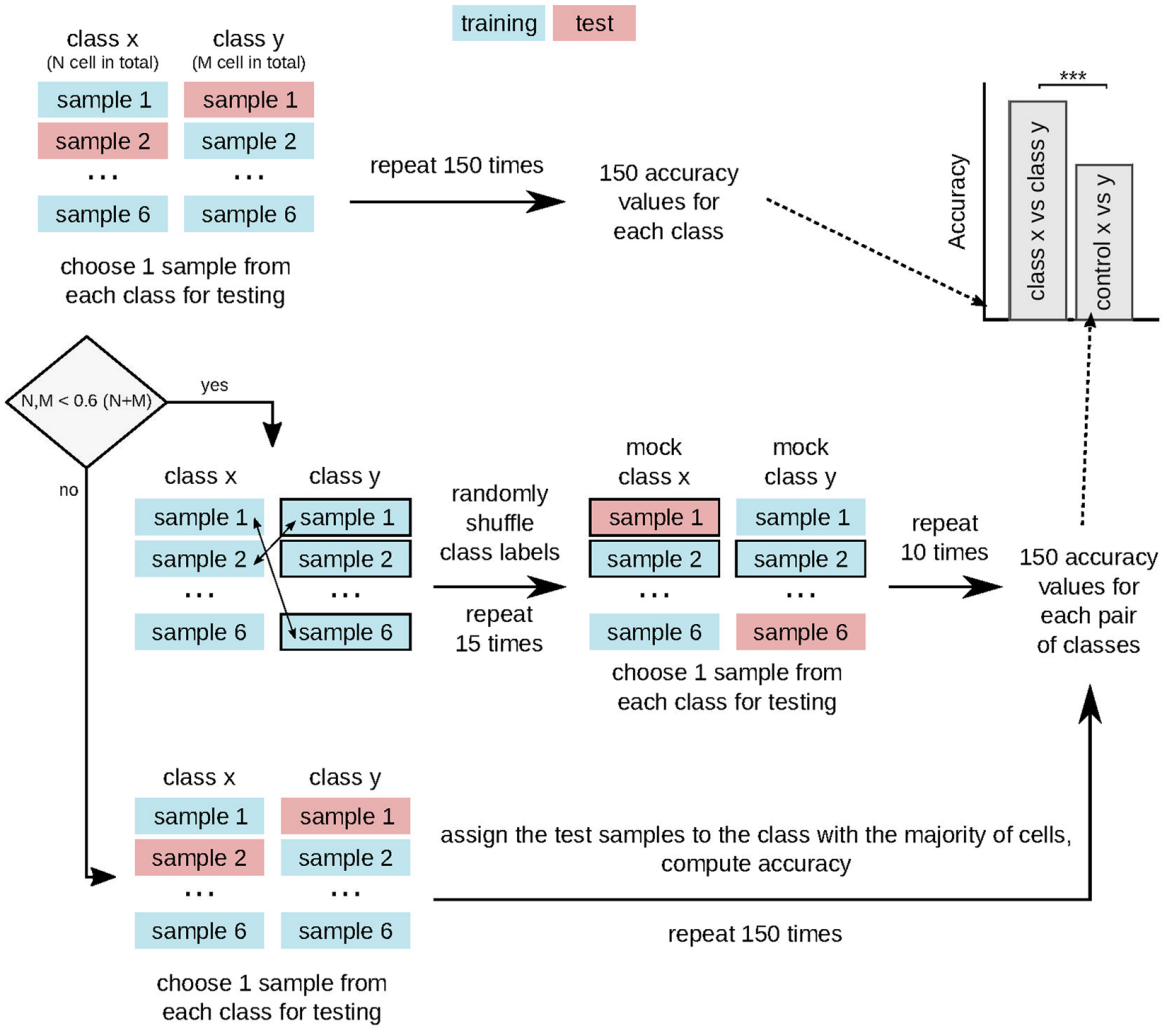
Schematic of the cross-validation scheme for experimental data. We compute the classifier accuracy for each pair of classes using two-class classification with stratified group shuffle split cross-validation. If the data is balanced (none of the classes contains more than 60% of cells), the control accuracy is computed by randomly shuffling the class labels and performing cross-validation with stratified group shuffle split. For unbalanced data, the majority classifier serves as a control.

The classifier accuracy was evaluated for each pair of classes by performing 150 rounds of cross-validation with stratified shuffle split for synthetic cells and stratified group shuffle split for T cells. For synthetic data, 50 cells from each class were randomly chosen in each cross-validation round to serve as the training dataset, while the remaining cells (20 cells per class) were used to test the classifier performance (Fig. 4). For experimental data, we had to combine the cells into groups according to time series (six groups per class). In each cross-validation round, one time series from each class was left out for testing, while the cells from the remaining time series served to train the classifier (Fig. 5). In this way, T cells from the same time series were either in the training or testing dataset, but not in both.

After evaluating the classifier accuracy, we compared it to a naive control classifier, which was chosen depending on whether the classified dataset was balanced or unbalanced. We considered a dataset to be balanced if none of the classes contained more than 60% of all cells. In this case, we used a random classifier as the best naive classifier. As a control for unbalanced data – where more than 60% of cells belonged to one class – we used the majority classifier. This classifier assigns all cells to the class with the most observation and thus performs better than a random classifier.

Since the dataset of synthetic cells was balanced (70 cells per class), we used a random classifier as control (Fig. 4). To compute the accuracy of the random classifier, we randomly shuffled class labels and carried out ten rounds of cross-validation with stratified-shuffle-split with 20 cells from each class serving as test data. We repeated the shuffling of class labels five times for each pair of classes, which produced 50 control accuracy values for each pair. We combined the accuracy values from all three pairs into a single control set with 150 values.

In contrast to synthetic data, each class of T cells contained different cell numbers (though the number of time series was the same). Hence, for each pair of classes, we determined whether the data was balanced or unbalanced and computed the control accuracy with either a random or a majority classifier (Fig. 5). For balanced data, we randomly shuffled class labels (repeated 15 times) and carried out cross-validation with stratified group shuffle split (10 rounds). For unbalanced data, we randomly selected one time series from each class (repeated 150 times), assigned all selected cells to the majority class, and computed the resulting accuracy. Thus, for each pair of classes, we used an individual control group with 150 accuracy values.

## Results

We used the described cross-validation scheme to classify migration patterns in both synthetic cells and T cells. We examined how the applied feature vector (static, dynamic time-domain, or dynamic frequency-domain features) affected the accuracy of the classifier. The classifier accuracy was further compared to that of a control classifier (random or majority) to identify experimental conditions and model parameters that significantly change the cell migration patterns.

### Dynamic SPHARM-based classification of synthetic cells

By detecting significant differences in migration patterns, our approach can be used to study how various parameters of the cell migration simulator (CMS) affect the shape of generated cells. To illustrate this use, we applied static and dynamic SPHARM-based classification to examine the behavior of the four parameters of the CMS. For each parameter, we examined three different values, while setting the other parameters to their default values. Thus, we tested the neighbor-weight parameter NW, which determines the cell surface roughness (default value NW = 4), the parameter for position weight PW, which affects the cell elongation (default value PW = 4), the distance weight parameter DW, which governs the size of cell protrusions (default value DW = 6), and the front-rear threshold FR, which is associated with the cell volume fraction considered as the front (default value FR = 0). We then quantified the classification accuracy between each pair of classes and compared it to a random control classifier to identify significant differences (Fig. 4).

**Figure 6:**
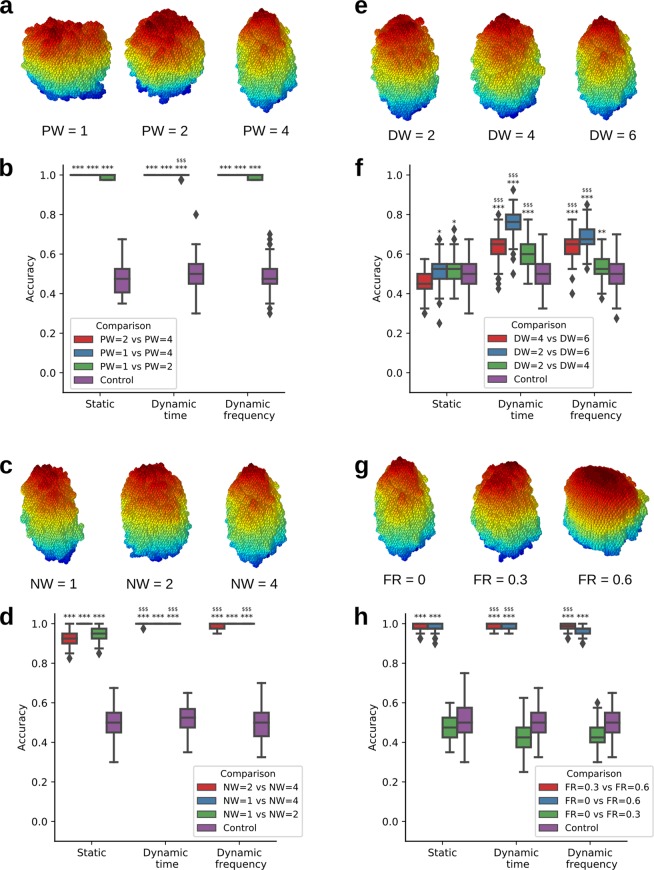
Classification of synthetic cells. Surfaces of representative cells generated with three different values of the position weight (PW) (**a**), neighbor weight (NW) (**c**), distance weight (DW) (**e**), and front-rear (FR) (**g**) parameters. Classifier accuracy for different pairs of classes relative to control for different values of the PW (**b**), NW (**d**), DW (**f**), and FR (**h**) parameters. Unless indicated, the default parameter values were used: FR = 0, NW = 4, PW = 4, DW = 6. Significantly higher than control: *p < 0.05, **p < 0.01, ***p < 0.001; significantly higher than static features: ^$$$^p < 0.001; one-sided Mann-Whitney test, n=150 per group.

Interestingly, different parameters affected cell shape to a different extent (Fig. 6). Thus, changing the value of the PW parameter from 1 to 2 to 4 resulted in visually distinct cells (Figs. 6a, S5a) and large quantitative differences between the cell shapes (Fig. 6b). Changing the value of NW from 1 to 2 to 4 resulted in a similarly large quantitative difference (Fig. 6d), but the cells’ 3D surface reconstructions were hard to distinguish visually (Fig. 6c). Only looking at the cells’ maximum projections revealed that – indeed – lower NW values resulted in a more structured cell surface (Fig. S5b). For the DW parameter, both 3D surfaces and maximum projections were similar (Figs. 6e, S5c) for different parameter values (2, 4, and 6), and no significant differences in cell shape could be identified by the static classifier (Fig. 6f). In contrast, when shape dynamics was included, the classifier accuracy was significantly higher than random, indicating that the visually similar cells might evolve their shape in different ways. Interestingly, different value ranges of the *FR* parameter had different effects on the cell shape, even though the parameter values were equally spaced. Thus, cell shape did not change when the value of *FR* increased from 0 to 0.3, which was both observed visually (Figs. 6g, S5d) and reflected by the close-to-random classifier accuracy (Fig. 6h). In contrast, cell shape for $FR=0.6$ was significantly different from both other classes, which was confirmed by visual inspection and by the classifier accuracy for all three feature vectors (Fig. 6g,h).

Although we examined only three values for each parameter, our approach should bring the most benefit when many parameter values and their combinations have to be scanned and it is hard to visually control the outcome of every combinations. Adjusting parameter values of a model can be a daunting task, and our approach can facilitate this endeavor by quantifying the shape differences that result from different parameter values and thus support the identification of meaningful parameter ranges. For instance, for our CMS, it would probably make little sense to further examine *FR* values between 0 and 0.3, since these values result in very similar cell shape, and one should rather focus on the *FR* values between 0.3 and 0.6.

When comparing the accuracy of the static and dynamic classifiers, dynamic features outperformed static features in nearly all comparisons (Fig. 6). No improvement was achieved only in those cases, where the static classifier accuracy was close to 100% (Fig. 6b,d), or the dynamic classifier detected no significant shape differences (Fig. 6h, $FR=0$.0 vs. $FR=0.3$). The use of dynamic frequency domain did not further improve classifier performance and resulted in similar accuracy values as for dynamic time domain (Fig. 6).

Importantly, the higher accuracy of the dynamic classifiers was not due to the larger dataset used to train them. As described in Materials and Methods, we used only the first time point for extracting static SPHARM features in order to keep the number of independent observations equal between the static and dynamic classifiers. Effectively, this resulted in a much larger feature vector for the dynamic classifiers while most of the information for the static classifier was discarded. Using all time points as independent observations, however, does not improve the performance of the static classifier because these data points are highly correlated. In fact, a static classifier based on all time points was inferior to the one based on just the first time point (Fig. S6). It also detected significant differences in cell shape where the actual classifier accuracy was only marginally higher than random (Fig. S6, $FR=0$ vs. $FR=0.3$). Thus, simply gathering cell shapes from all time points into a static classifier not only doesn’t help to improve the classifier accuracy, but also can result in overfitting and misleading conclusions. Only a truly dynamic way to handle time information can boost the classifier performance by including the shapes at different time points into feature vectors of individual cells.

### Dynamic SPHARM-based classification of T cells

We further examined whether our approach can also be used to classify migration patterns in experimental data. We analyzed the three groups of T cells migrating in various tissues (LN, skin, and SMG). As in synthetic data, we quantified the classification accuracy between each pair of tissues and compared it to a control classifier (Fig. 5).

**Figure 7:**
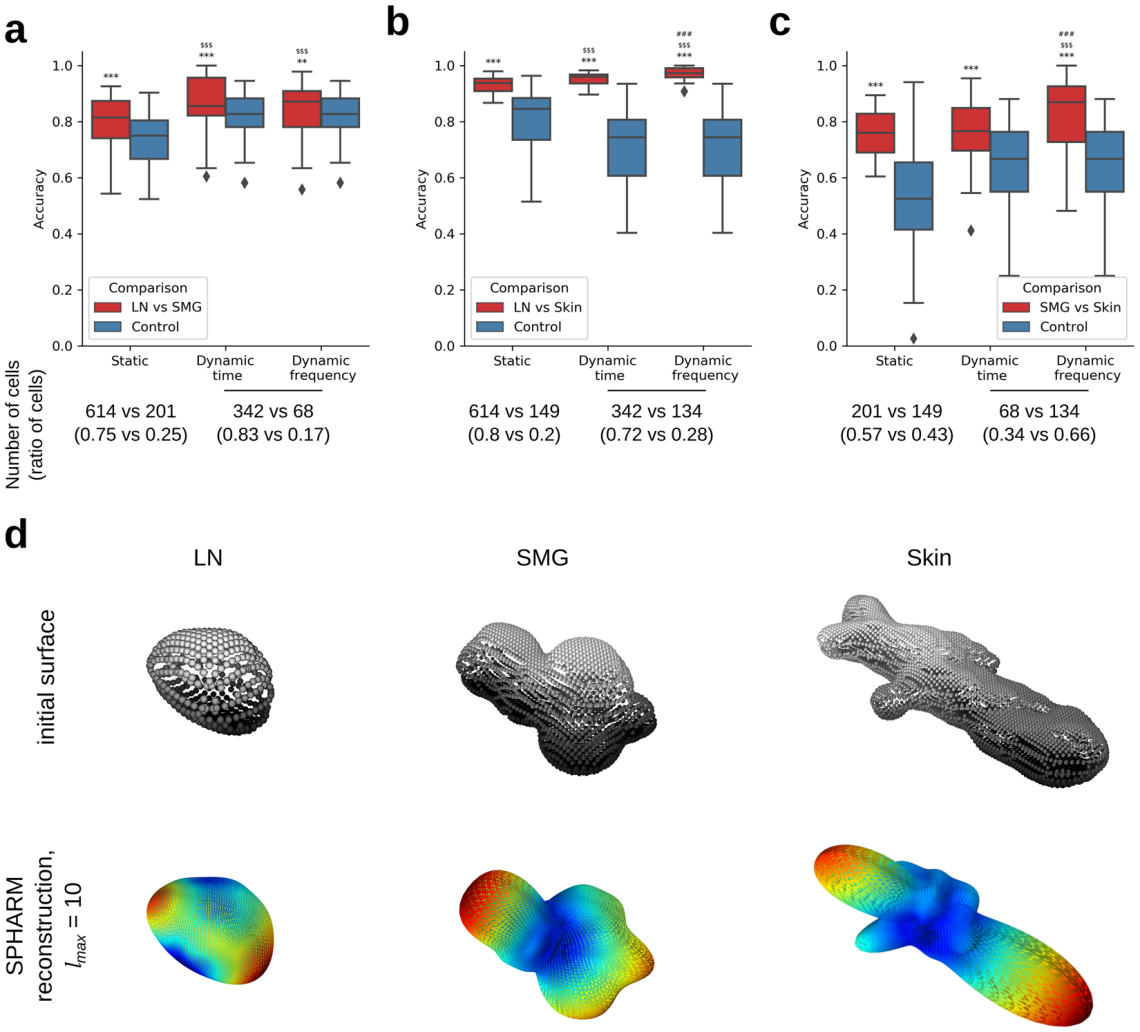
Classification of T cells for various pairs of classes relative to control. (**a**) LN vs SMG. (**b**) LN vs Skin. (**c**) SMG vs Skin. (**d**) Surfaces and SPHARM reconstructions of representative cells from the three analyzed classes. Significantly higher than control: **p < 0.01, ***p < 0.001; significantly higher than static features: ^$$$^p < 0.001; significantly higher than dynamic time features: ^###^p < 0.001; one-sided Mann-Whitney test, n = 150 per group. Numbers below the box plots indicate the number (ratio) of cells in each class for the corresponding class pair. For ratios between 0.4 and 0.6, the random classifier was used as control. In all other cases, the majority classifier was used, whose accuracy is defined by the ratio of cells. In the dynamic classifiers, fewer cells were analyzed because not all cell tracks were sufficiently long (20 time points) to be included. This resulted in different ratios between cell numbers and hence different control accuracies for the static and dynamic classifiers.

The classifier accuracy significantly differed from control for each comparison and each feature vector (Fig. 7a–c). This difference was most apparent for the comparison of environments LN versus skin (Fig. 7b) because these tissues induce two extremes in the shape of T cells (Figs. 2c, 7d). The T cells migrating in LN had a more regular close-to-spherical shape, whereas the T cells from skin were irregular and made many protrusions. T cells migrating in SMG had an intermediate shape, which also strongly varied from time series to time series.

The relative accuracy of the static and dynamic classifiers differed for each comparison, but the dynamic classifiers generally performed better (Fig. 7a–c). Thus, the dynamic classifiers both in the time- and frequency-domain were superior when comparing LN to the other two environments, whereby in the LN vs. skin comparison, the accuracy further increased with the use of dynamic frequency features. When comparing SMG and skin, the use of dynamic time features did not bring any benefit, whereas the dynamic frequency features outperformed both dynamic time and static features. As a common trend for all comparisons, the classifier accuracy with dynamic frequency features was always significantly higher than the accuracy of the static classifier. This trend confirms our hypothesis that not only static shape features but also the dynamic shape patterns play an essential role in defining unique modes of cell migration in different environments.

### Comparison of SPHARM-based classification to 2D shape descriptors

We evaluated how SPHARM-based descriptors perform in comparison to 2D shape descriptors. As SPHARM is a 3D extension of a Fourier analysis, we applied a method that is based on the classification of a set of Fourier components produced by Discrete Fourier Transform (DFT) after carrying out a 3D-to-2D projection of surface-rendered cells^35^. The advantage of the latter approach is two-fold: (i) the more complex 3D shape characterization by SPHARM is reduced to 2D DFT and (ii) the combination of various random 3D-to-2D projections allows a seamless transition from a pure 2D shape descriptor to a quasi-3D description of cell shapes. We combined these 2D descriptors obtained from various numbers of projections with the same classification scheme that we used for SPHARM and compared the classification accuracy to a static SPHARM classifier.

**Figure 8:**
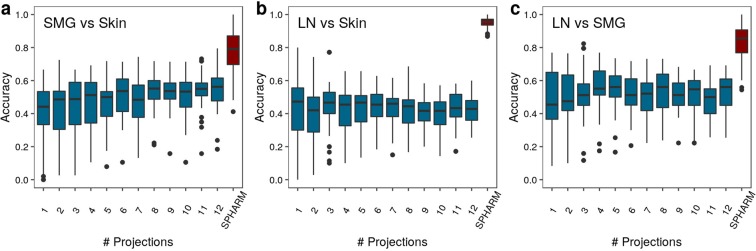
Static SPHARM-based classifier accuracy for T cells of different pairs of environments in comparison to the DFT-based with projection numbers between 1 and 12. The environments. (**a**) SMG versus Skin, (**b**) LN versus Skin, and (**c**) LN versus SMG are compared.

The results of this comparison for synthetic cells are summarized in Figs. S7–S10 and reveal that the SPHARM-based approach always outcompetes the 2D shape descriptors, except for variations in the DW parameter (see Fig. S9) and for a specific comparison of FR parameters (see Fig. S10a). This does not come as a surprise, because we noted before that for these parameters the SPHARM-based classifier does not perform better than the random classifier in the static case (see Fig. 6f,h). These observations suggest that the DFT-based approach is more sensitive to details in the outline of the shape projections than the SPHARM-based approach. On the other hand, increasing the number of projections gives rise to a quasi-3D description of cell shapes that is approaching but not superseding the accuracy of the SPHARM-based classification for variations in the model parameters PW, NW and FR. For T cells, the performance of the static SPHARM-based classification compared with the DFT-based revealed that the accuracy of the former is always significantly higher, even if the latter approach includes numerous projections for a quasi-3D representation of the cell shapes. In fact, as can be seen in Fig. 8, the classification accuracy of the DFT-based approach is comparable to that of a random classifier. We conclude that the complexity of the T cell shape determines the minimum number of projections for which the DFT-based approach of T cell representation performs as well as the SPHARM-based approach does by 3D reconstruction.

We conclude from this comparison that the SPHARM-based approach is generally superior to using 2D shape descriptors.

## Discussion

In our proof-of-principle study, we confirmed that adding dynamics to shape analysis can improve the classification of migration patterns of cells. We illustrated this improvement by combining the dynamic SPHARM approach with an SVM classifier to identify conditions with significantly different cell shapes. This approach, however, is not restricted to SPHARM and SVM. We believe that adding dynamics to other shape descriptors, such as Fourier-based descriptors^35^ or wavelets^36,37^, could also improve their classification accuracy. The performance of other classifiers – such as random forest or neural networks – should also be studied in the future.

Other implementations of the SPHARM analysis should also be tested, e.g. the SPHARM-MAT toolbox available in MATLAB. Here we focused on an open-source Python alternative SHTools^31^ making this analysis freely available to the community. The problem of the SHTools implementation, however, is that it is based on converting the cell shape to spherical coordinates and is therefore accurate only for cells with the center of mass inside the cell body. While this requirement was always met for synthetic cells, some T cells – especially those migrating in the skin – had a complex shape with the center of mass temporarily appearing outside the cell body. Such shape could not be accurately represented in spherical coordinates, which resulted in erroneous shape reconstructions after SPHARM transform (Fig. S11, bottom row). Since such complex-shaped cells constituted only a fraction of the total T cell number (9% LN, 7% in SMG, and 28% in the skin), we neglected this inaccuracy in the current study, but this problem will have to be addressed in the future. One solution to this could be to create open-source implementations of other SPHARM variants (like SPHARM-MAT) that calculate the SPHARM transform directly from the Cartesian coordinates of the surface and thus do not suffer from the center-of-mass problem.

Despite the limitations of our approach for cells with highly irregular shape, most of the cells analyzed in our study (all synthetic cells, as well as> 90% of LN and SMG T cells) had their center of mass inside the cell and thus could be accurately analyzed by our method. In all comparisons that involved these accurately represented cells (Figs. 6, 7a), dynamic SPHARM outperformed its static counterpart and – in one case – could even reveal shape differences not detected by the static SPHARM analysis (Fig. 6f). This suggests that including dynamic shape features is especially relevant when shape differences between the analyzed groups are small, whereas for visually distinct shapes, static analysis – or even simpler shape descriptors – may be sufficient.

When looking at dynamic SPHARM in the frequency domain, in most cases it did not bring any improvement relative to the time domain. Interestingly, the only comparisons where the dynamic frequency features were superior involved the T cells migrating in skin (Fig. 7b,c), which included a relatively high fraction (28%) of cells with center of mass transiently appearing outside the cell body. This event resulted in a dramatic change in shape representation for some of the time points (Fig. S11, bottom row) and apparently created a unique frequency pattern that was picked up by the dynamic frequency classifier and used as a feature to distinguish skin T cells from other T cells. This observation confirms that the use of frequency domain can indeed help to detect interesting frequency patterns^8^, either relevant for cell migration, or irrelevant artifacts as in our case.

By recognizing differences in dynamic cell shape, dynamic shape analysis methods like ours can be used to study how cell migration patterns change under various experimental conditions. Here we showed that the extracellular environment affects the dynamic shape of migrating T cells: even though we looked at the same type of cells, their migratory behavior dramatically changed depending on the surrounding tissue. In the future, a similar approach can be used to study other factors that affect cell migration^1^ (Fig. 1), which could involve various cell types migrating in different intact or altered extracellular environments both *in vivo* and in 3D artificial matrices^38^.

Dynamic SPHARM analysis can also be applied to study cell migration *in silico*. Classifying between cells generated with different parameters of migration models will help to better understand the meaning of these parameters both in our CMS and other models of cell migration. Furthermore, comparing the shape of synthetic migrating cells to that of real cells will enable the use of the image-based systems biology approach^39,40^ to create cell migration models that are realistic and data-driven. With these models, we can identify and interpret the meaning of the model parameters that induce cell shapes observed in a particular experiment^41^.

Taken together, dynamic SPHARM-based classification can contribute to our understanding of cell migration in two ways. On the one hand, it can help to investigate various experimental settings and their effect on cell migration patterns. On the other hand, it should enable identifying parameters of realistic migration models that produce specific experimentally observed cell shapes. Both of these applications can help to pinpoint the factors that affect the shape of migrating cells and thus bring us closer to understanding the mechanisms underlying cell migration.

## Supplementary information

supplementary information.

supplementary information 2.

supplementary information 3.

supplementary information 4.

supplementary information 5.

supplementary information 6.

supplementary information 7.

## Data Availability

The authors declare that all relevant data supporting the findings of this study are available within the paper (and its Supplementary Information file). Any raw data can be downloaded from https://asbdata.hki-jena.de/publidata/MedyukhinaEtAL_SPHARM/. The source code for the dynamic SPHARM analysis is available at https://github.com/applied-systems-biology/Dynamic_SPHARM. The source code for the cell migration simulator is available at https://github.com/applied-systems-biology/cell-migration-simulator.
