# Supplementary material for: Dynamic spherical harmonics approach for shape classification of migrating cells
Source: Sci Rep. 2020 Apr 8;10:6072. doi: 10.1038/s41598-020-62997-7 (PMC7142146; doi:10.1038/s41598-020-62997-7)
Supplement: Supplementary file 1 — supplementary information. [file 41598_2020_62997_MOESM1_ESM.pdf]

Figure S1

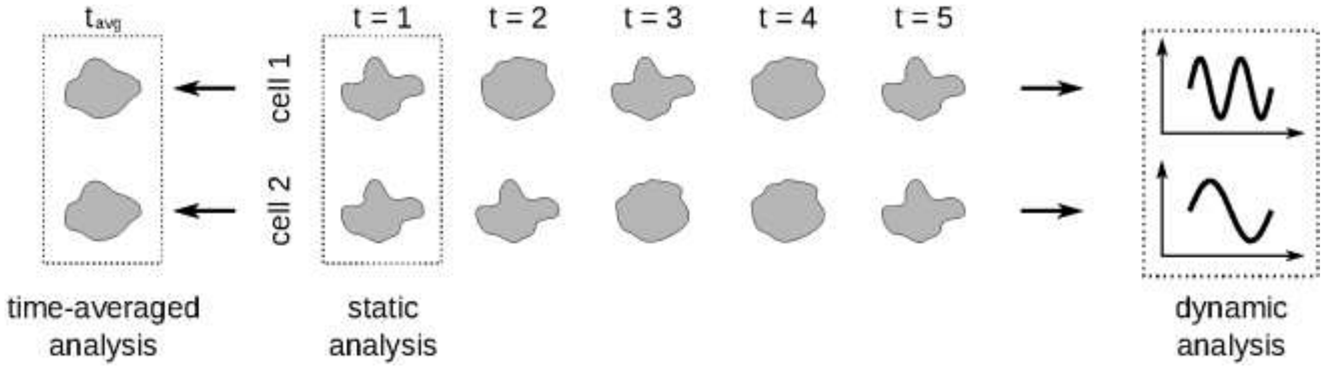

Figure S1: Dynamic shape analysis can reveal patterns that are obscured in static or time-averaged analyses.

Figure S2

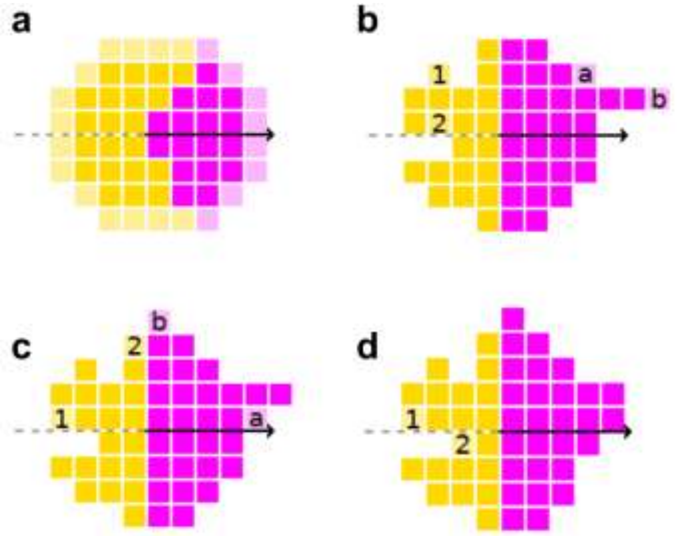

Figure S2: Schematic overview of the parameters of the migration model. (a) Example of a cell with the FR (front-rear threshold) parameter > 0; for $FR = 0$, see (b)-(d) and Fig. 1a. (b) Impact of the number of neighbors; removing SU 1 with one neighbor is more likely than removing SU 2 with three neighbors (von Neumann neighborhood); moving the SU into position a with two neighbors is more likely, than into position b with one neighbor. (c) Impact of the position vector; SU 1 is located closer to the migration axis and therefore has a higher probability of being removed than SU 2; position a is preferred over position b to place the new SU because it is closer to the migration axis. (d) Impact of the distance to the cell's center of mass; SU 1 is located further away from the center of mass and therefore has a higher probability of being removed than SU 2.

Figure S3

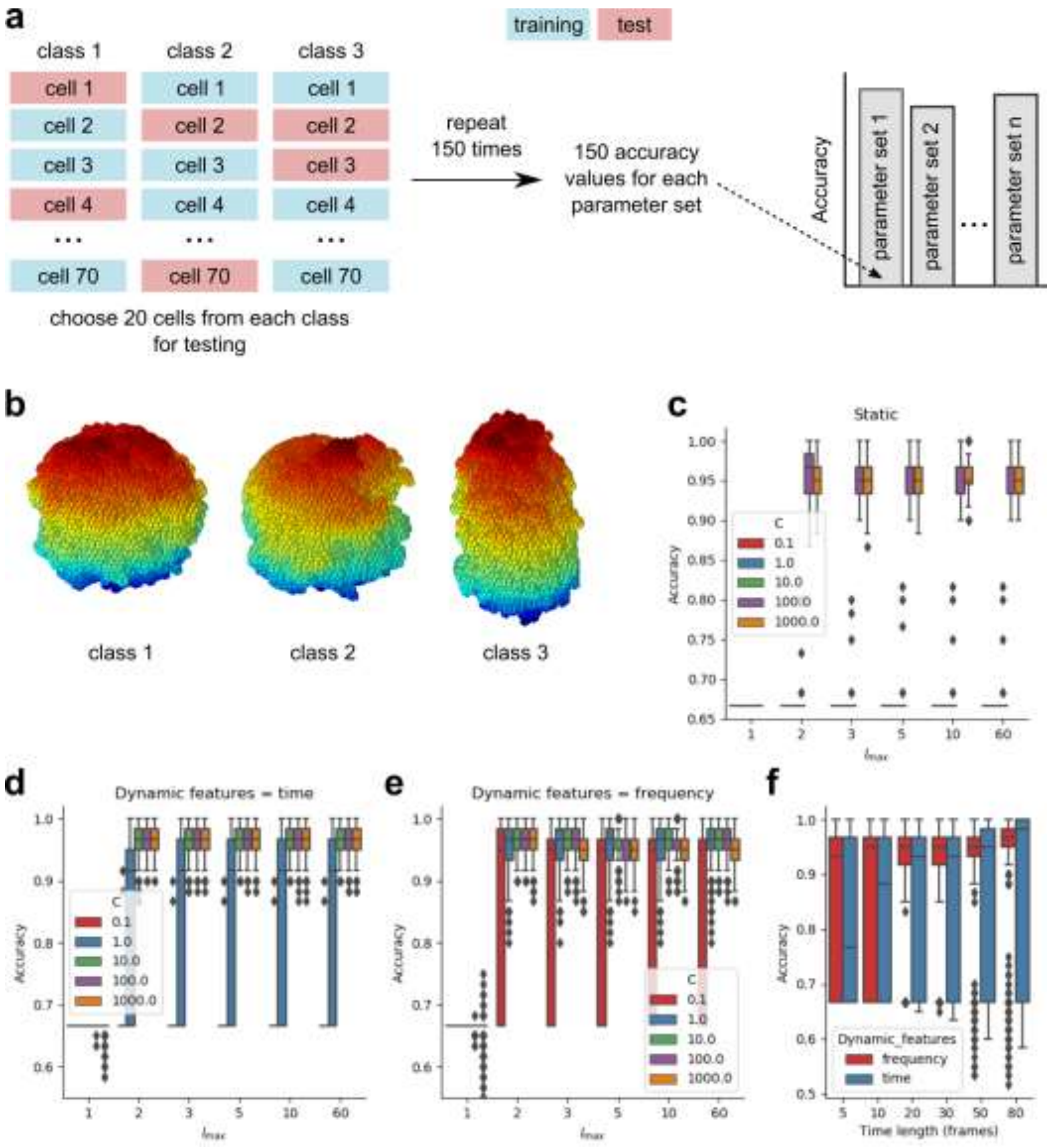

Figure S3: Adjusting classification parameters for synthetic cells. (a) We compute the classifier accuracy for each set of parameters using three-class classification with stratified shuffle split cross-validation. (b) Representative cells from the three analyzed classes. (c) Accuracy of the static classifier for different values of the l_{max} and C parameters. (d) Accuracy of the dynamic time classifier for different values of l_{max} and C. (e) Accuracy of the dynamic frequency classifier for different values of l_{max} and C. (f) Accuracy of both dynamic classifiers for all values of C and l_{max} depending on the number of analyzed time points.

Figure S4

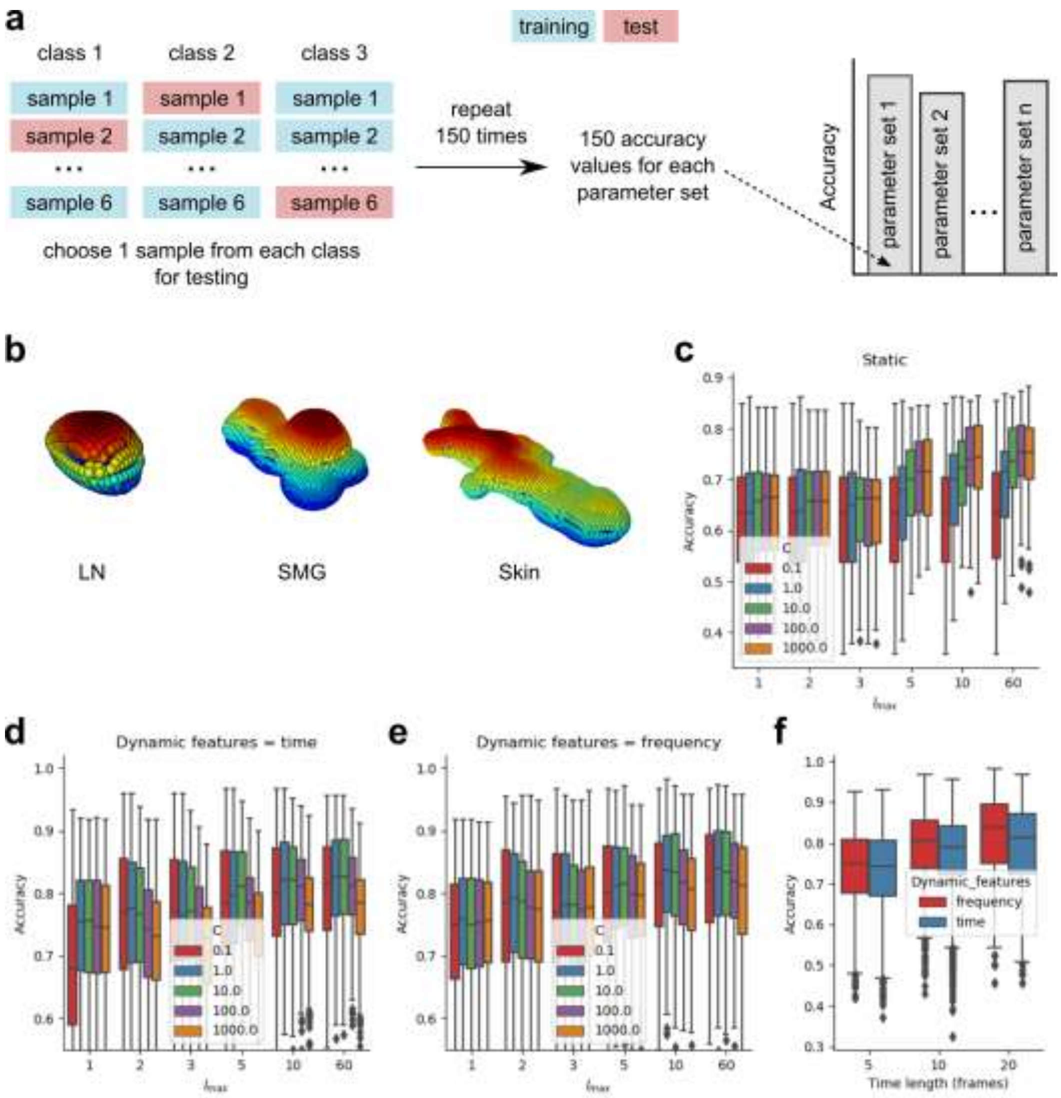

Figure S4: Adjusting classification parameters for T cells. (a) We compute the classifier accuracy for each set of parameters using three-class classification with stratified group shuffle split cross-validation. (b) Representative cells from the three analyzed classes. (c) Accuracy of the static classifier for different values of the l_{max} and C parameters. (d) Accuracy of the dynamic time classifier for different values of l_{max} and C. (e) Accuracy of the dynamic frequency classifier for different values of l_{max} and C. (f) Accuracy of both dynamic classifiers for all values of C and l_{max} depending on the number of analyzed time points.

Figure S5

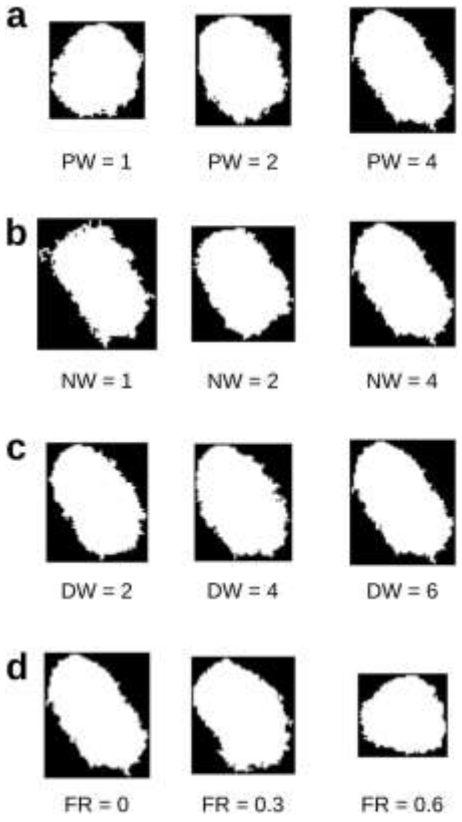

Figure S5: Maximum projections of representative cells generated with three different values of the position weight (PW) (a), neighbor weight (NW) (b), distance weight (DW) (c), and front-rear (FR) (d) parameters. Unless indicated, the default parameter values were used: FR = 0, NW = 4, PW = 4, DW = 6

Figure S6

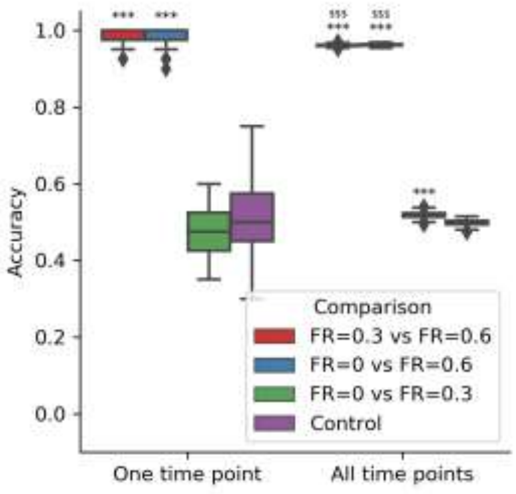

Figure S6: Static classifier accuracy for different pairs of classes relative to control for different values of the FR parameter. Classification was done using either only one (the first) time point of each cell track or all time points. For the parameters other than FR, default values were used: NW = 4, PW = 4, DW = 6. Significant difference from control: *** p<0.001; significant difference from the one-time-point classifier: $$$ p<0.001; two-sided Mann-Whitney test.

Figure S7

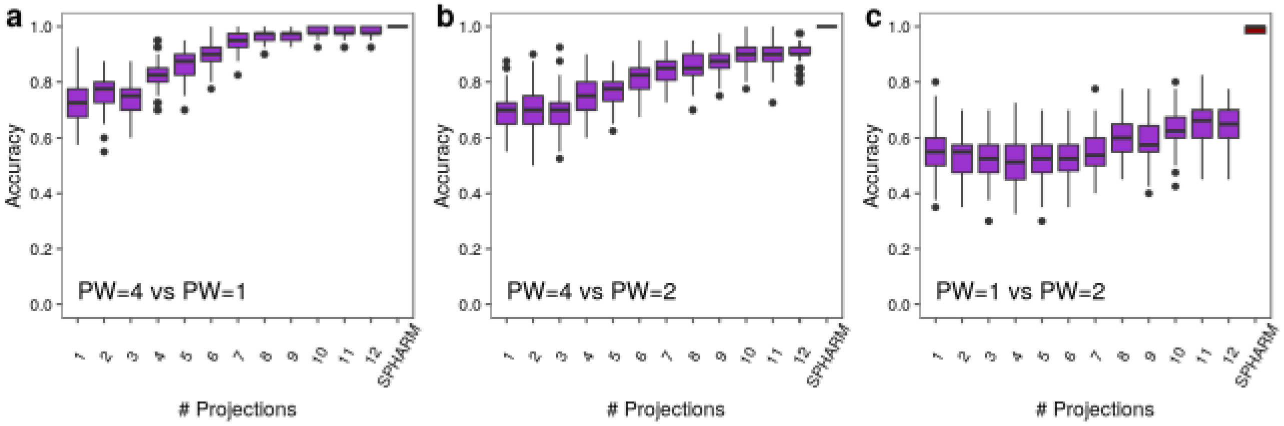

Figure S7: Static SPHARM-based classifier accuracy for synthetic cells of different pairs of classes of the PW parameter in comparison to the DFT-based with projection numbers between 1 and 12. The classes (a) PW=4 versus PW=1, (b) PW=4 versus PW=2, and (c) PW=1 versus PW=2 are compared. For the parameters other than PW, default values were used: NW = 4, FR = 0.0, DW = 6.

Figure S8

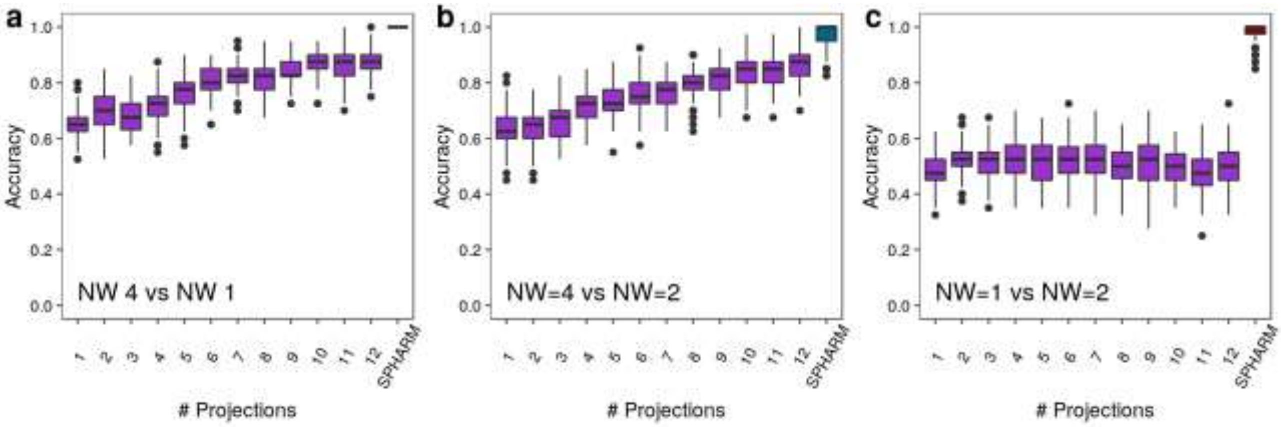

Figure S8: Static SPHARM-based classifier accuracy for synthetic cells of different pairs of classes of the NW parameter in comparison to the DFT-based with projection numbers between 1 and 12. The classes (a) NW=4 versus NW=1, (b) NW=4 versus NW=2, and (c) NW=1 versus NW=2 are compared. For the parameters other than NW, default values were used: PW = 4, FR = 0.0, DW = 6.

Figure S9

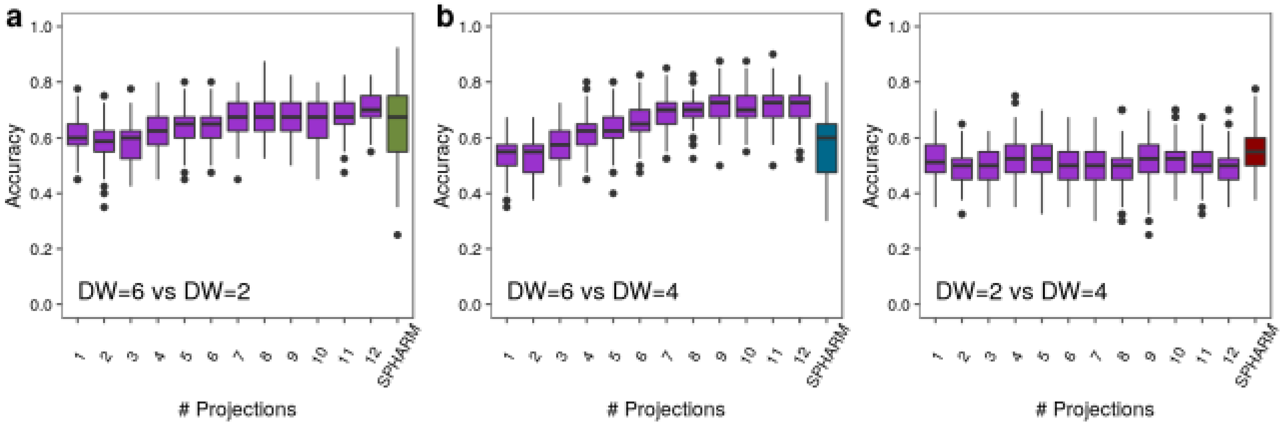

Figure S9: Static SPHARM-based classifier accuracy for synthetic cells of different pairs of classes of the DW parameter in comparison to the DFT-based with projection numbers between 1 and 12s. The classes (a) DW=6 versus DW=2, (b) DW=6 versus DW=4, and (c) DW=2 versus DW=4 are compared. For the parameters other than DW, default values were used: PW = 4, FR = 0.0, NW = 4.

Figure S10

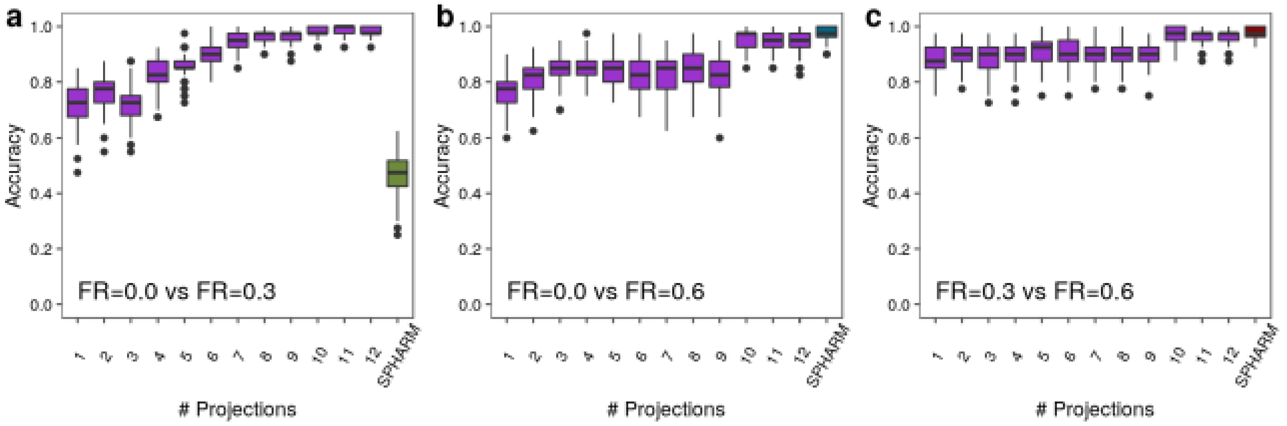

Figure S10: Static SPHARM-based classifier accuracy for synthetic cells of different pairs of classes of the FR parameter in comparison to the DFT-based with projection numbers between 1 and 12. The classes (a) FR=0.0 versus FR=0.3, (b) FR=0.0 versus FR=0.6, and (c) FR=0.3 versus FR=0.6 are compared. For the parameters other than FR, default values were used: PW = 4, DW = 6, NW = 4.

Figure S11

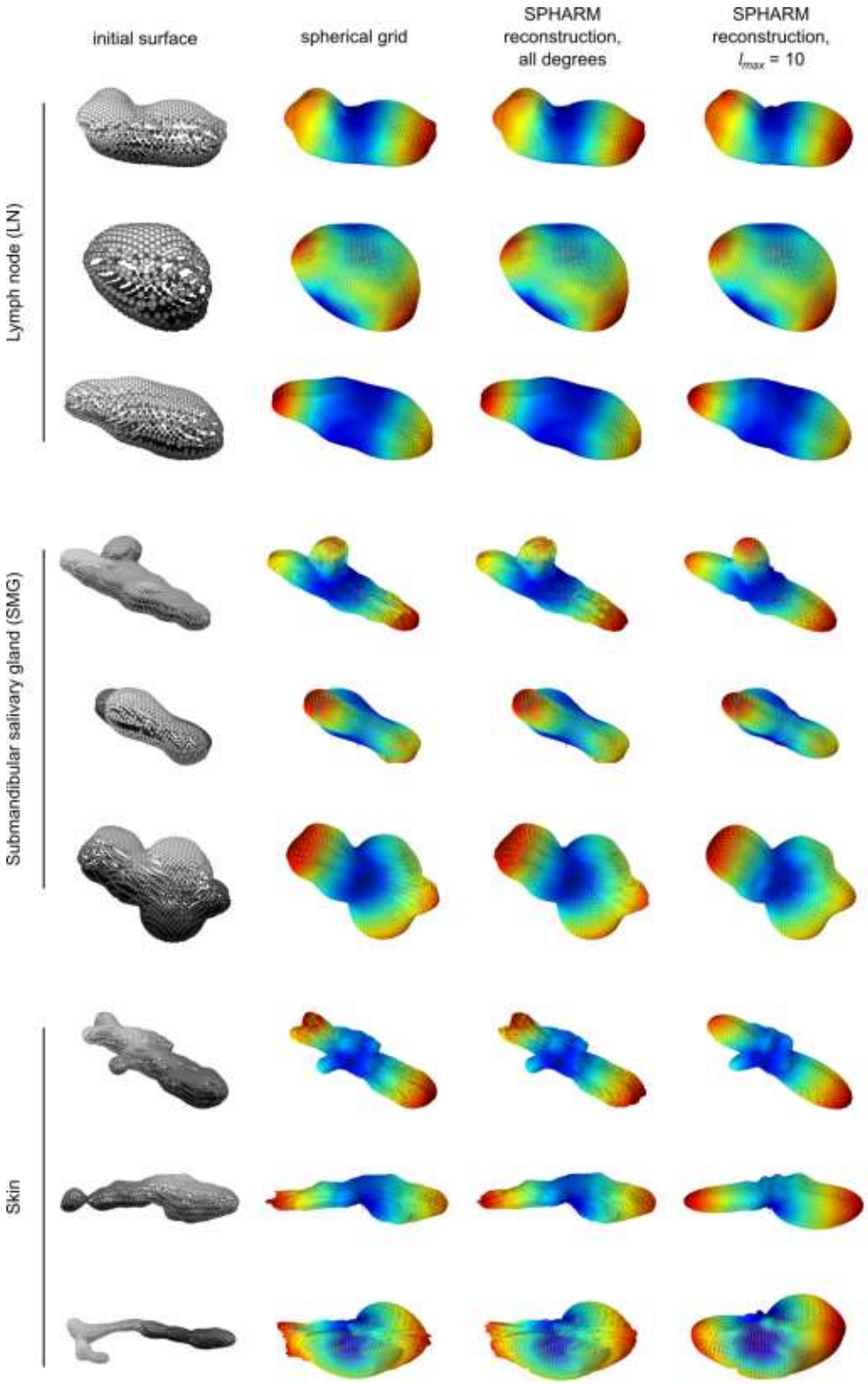

Figure S11: Examples of T cell surfaces before and after interpolating them onto a regular spherical grid, as well as after an inverse SPHARM transform.
