# Supplementary material for: Dynamic spherical harmonics approach for shape classification of migrating cells
Source: Sci Rep. 2020 Apr 8;10:6072. doi: 10.1038/s41598-020-62997-7 (PMC7142146; doi:10.1038/s41598-020-62997-7)
Supplement: Supplementary file 2 — supplementary information 2. [file 41598_2020_62997_MOESM2_ESM.pdf]

Supplementary methods

Dynamic spherical harmonics approach for shape classification of migrating cells

Anna Medyukhina[1, ‡,]†, Marco Blickensdorf[1,]†, Zoltán Cseresnyés[1], Nora Ruef[3], Jens V. Stein[3], Marc Thilo Figge[1,2,4,]*

[1]Applied Systems Biology, Leibniz Institute for Natural Product Research and Infection Biology – Hans Knöll Institute (HKI), Jena, Germany.

[2]Institute of Microbiology, Faculty of Biological Sciences, Friedrich Schiller University Jena, Jena, Germany.

[3]Department of Oncology, Microbiology and Immunology, University of Fribourg, Fribourg, Switzerland.

[4]Center for Sepsis Control and Care (CSCC), Jena University Hospital, Jena, Germany.

‡ This author's current affiliation is Center for Bioimage Informatics, St. Jude Children's Research Hospital, Memphis, TN

† These authors contributed equally to this work.

* Correspondence should be addressed to M.T.F.: thilo.figge@leibniz-hki.de

Experimental data

Mice

OT-I TCR[1] were backcrossed to Tg(UBC-GFP)30Scha "Ubi-GFP"[2] or hCD2-dsRed[3] mice. 6-10 week-old sex-matched C57BL/6 mice (Janvier, France) were used as recipient mice. All mice were maintained at the animal facility of the Department of Clinical Research/University of Bern or at the University of Fribourg. All animal work has been approved by the Cantonal Committee for Animal Experimentation and conducted according to federal guidelines.

T cell transfer and viral infections

$CD8^+$ T cells were negatively isolated from spleen and peripheral lymph nodes of GFP^+ or $dsRed^+$ OT-I, using the EasySep Mouse $CD8^+$ T Cell Isolation Kit (Stem Cell Technologies). OT-I T cells (5×10^4) were i.v. transferred into recipient mice 24 h before i.p. infection with 10^5 pfu LCMV-OVA[4]. For skin imaging, 15 µl 0.3% DNFB (in acetone/oil 4:1) was applied to the right flank on day 3 p.i. and 500 ng SIINFEKL peptide was applied on days 4 and 5 p.i.. Two-photon microscopy (2PM) imaging was performed ≥ 30 days p.i..

2PM image acquisition and analysis

2PM intravital imaging of the popliteal lymph node, SMG, and skin was performed as described [5,6]. In brief, mice were anesthetized with ketamine/xylazine/acepromazine, and for lymph node (LN) imaging the right popliteal lymph node was surgically exposed. Before recording, Alexa 633-conjugated MECA-79 (10 µg/mouse) was injected i.v. to label HEV. For submandibular salivary gland (SMG) imaging, the right SMG lobe was surgically exposed. For skin imaging, a section of the right flank skin was elevated onto a metal holder by making two parallel incisions.

2PM imaging was carried out with a TrimScope 2PM system (LaVision Biotec) using a 25X Nikon (NA 1.0) objective. ImSpector software was used to control the 2PM system and acquire

images. Images were obtained with the help of an automated system providing real-time drift correction [7]. Excitation was provided by a Ti:sapphire laser (Mai Tai HP or DeepSee, Spectra-Physics) tuned to 780, 840, 900 or 940 nm in combination with 1045 nm. 10 to 26 x-y slices with a z-step size of 2-4 µm were acquired in 0-140 µm depth. The time interval was 20 s for LN and SMG and 60 s for the skin and the imaging time was 20-60 minutes. Emitted light and second harmonic signals were detected through 447/55-nm, 525/50-nm, 593/40-nm and 655/40-nm bandpass filters with non-descanned photomultipliers.

Image preprocessing

Two-photon intravital images were provided as OME-TIFF images, which were converted to single time point and single Z layer TIFF images using a custom-written ImageJ macro. These images were loaded into HuygensPro 19.04 (SVI, Hilversum, Holland) in order to be deconvolved. The deconvolution process was carried out by using the theoretical point spread function (PSF), as provided by HuygensPro's Classic Maximum Likelihood Estimation method, based on the Richardson-Lucy algorithm. The deconvolved images were saved as multilayer TIFF files, one image stack per time point.

T-cell segmentation and tracking

The deconvolved time-series images were loaded into Imaris 9.3.1 (Bitplane, Zürich, Switzerland) for further processing. In the first step, the image stacks were thresholded with the Otsu algorithm in order to identify the voxels that belonged to the foreground, representing the fluorescently labeled T-cells. Typically the threshold values allowed the fluorescence range of 1000 to 15000 to be considered as part of the reconstructed cell surface. The T-cells were then segmented as 3D surfaces using the Surfaces object-creation wizard of Imaris. The surface rendering was adjusted to the complex cell shape of the active T-cells by setting the smoothing scale to 0.2 micrometers and the local threshold search radius to 1.0 micrometer, in order to make the Surfaces objects fit the volume image of the T-cells precisely. Objects below the volume of 1500 voxels were eliminated, in order to avoid detecting debris.

In the next step of the analysis, the reconstructed surfaces that represented individual T-cells were tracked. For this, the Autoregressive Motion tracking method of Imaris was applied, with a maximum allowed gap size of one time step, and a minimum track length corresponding to 50% of the total duration of the experiment. Each time series of the reconstructed and tracked images were individually observed in the next step of the analysis, in order to identify potential tracking errors caused by multiple T-cells forming a temporary cluster that would have been identified as one large cell. Such artificial clusters were manually removed from further analysis. These corrections were especially crucial for the skin samples, where the T-cells often assumed a very complex shape.

The tracked and manually checked T-cell surfaces were exported from Imaris, using the VRML (Virtual Reality Modeling Language) format. These .wrl files described each T-cell surface at every time point of the experiment. The cell tracks were exported as Excel .xls files. The .wrl files were parsed by a customized Python script to extract the surface coordinates of individual cells, which were then combined into time-series with the help of the track files.

Adjusting the classification parameters

Before applying the classifier to distinguish between types of migrating cells, several parameters of the classification and the feature extraction workflow had to be adjusted. First, we needed to choose the number of SPHARM degrees l_{max} used in the analysis. Second, for the dynamic analysis, we needed to select the number of analyzed time points T. Finally, the penalty parameter C of the SVM classifier had to be adjusted to obtain the optimal classification results. Whereas the first two parameters (l_{max} and T) determine the number of features used for classifications, parameter C defines how well classification errors are tolerated by SVM while fitting the training data. With small values of C, more errors are tolerated, whereas, with large C values, SVM tries to classify the training dataset with as high accuracy as possible.

To choose the optimal values of these parameters for synthetic data, we generated a synthetic dataset of 210 cells from three visually distinct classes (Fig. S2b). For each parameter combination, we performed 100 rounds of cross-validation with stratified shuffle split, where 5/7 of the data (50 cells from each class) were randomly chosen as the training set, and 2/7 (20 cells from each class) served as the test set (Fig. S2a). The accuracy of the classifier was evaluated for all parameter combinations and all three feature vectors (Fig. S2c-f), and the parameters that provided the highest accuracy for all three feature vectors were chosen for further analysis (l_{max}=2, C=100, T=80).

The parameter adjustment for T cells was performed similarly, but the stratified shuffle split was replaced by stratified group shuffle split, where one time-series from each class was used for testing and the rest (five time-series) were used for training (Fig. S3a). Similarly to synthetic cells, cross-validation was carried out for 100 rounds for each parameter combination, and the accuracy of the classifier was quantified for both static and dynamic features (Fig. S3c-f). Due to the limited duration of the T cell tracks, we had to use fewer time points than in synthetic data (T=10). Also, a higher value of l_{max} (l_{max}=10) had to be used, likely in order to increase the size of the feature vector and thus to compensate for the small value of T. For the C parameter, different values were optimal for the static and dynamic analysis, and therefore C=100 was chosen for the static classifier and C=10 was chosen for both dynamic classifiers.

References

1. Hogquist, K. A. *et al.* T cell receptor antagonist peptides induce positive selection. *Cell* (1994). doi:10.1016/0092-8674(94)90169-4

2. Schaefer, B. C., Schaefer, M. L., Kappler, J. W., Marrack, P. & Kedl, R. M. Observation of antigen-dependent CD8+ T-cell/dendritic cell interactions in vivo. *Cell. Immunol.* (2001). doi:10.1006/cimm.2001.1895

3. Kirby, A. C., Coles, M. C. & Kaye, P. M. Alveolar Macrophages Transport Pathogens to Lung Draining Lymph Nodes. *J. Immunol.* (2009). doi:10.4049/jimmunol.0901089

4. Kallert, S. M. *et al.* Replicating viral vector platform exploits alarmin signals for potent CD8+ T cell-mediated tumour immunotherapy. *Nat. Commun.* (2017). doi:10.1038/ncomms15327

5. Ficht, X., Thelen, F., Stolp, B. & Stein, J. V. Preparation of murine submandibular salivary gland for upright intravital microscopy. *J. Vis. Exp.* (2018). doi:10.3791/57283

6. Moalli, F. *et al.* The Rho regulator Myosin IXb enables nonlymphoid tissue seeding of protective CD8+ T cells. *J. Exp. Med.* (2018). doi:10.1084/jem.20170896

7. Vladymyrov, M., Abe, J., Moalli, F., Stein, J. V. & Ariga, A. Real-time tissue offset correction system for intravital multiphoton microscopy. *J. Immunol. Methods* (2016). doi:10.1016/j.jim.2016.08.004
