# Supplementary material for: Dynamic spherical harmonics approach for shape classification of migrating cells
Source: Sci Rep. 2020 Apr 8;10:6072. doi: 10.1038/s41598-020-62997-7 (PMC7142146; doi:10.1038/s41598-020-62997-7)
Supplement: Supplementary file 3 — supplementary information 3. [file 41598_2020_62997_MOESM3_ESM.pdf]

Video S1: Example of a cell generated with our cell migration simulator.

Video S2: $CD8^+$ T cells migrating in a lymph node. The deconvolved volume images and the reconstructed Surfaces objects from Imaris were animated as a time series at 15 fps. The beginning of the video shows the volume signal alone, followed by a linear transition into non-transparent Surfaces view by the time series reaches the halfway point. The video then fades the Surfaces objects down to 57% transparency by the end of the image series. The video then resets to the original volume view as seen at zero time point. The view of the video is set at 45 degrees perspective. The scale bar indicates the calibrated size of the scene.

Video S3: $CD8^+$ T cells migrating in a submandibular salivary gland. For details of video creation see the caption of Video S2.

Video S4: $CD8^+$ T cells migrating in skin. For details of video creation see the caption of Video S2.
